# Diastolic Augmentation Index Improves Radial Augmentation Index in Assessing Arterial Stiffness

**DOI:** 10.1038/s41598-017-06094-2

**Published:** 2017-07-19

**Authors:** Yang Yao, Liling Hao, Lisheng Xu, Yahui Zhang, Lin Qi, Yingxian Sun, Benqiang Yang, Frans N. van de Vosse, Yudong Yao

**Affiliations:** 1 Sino-Dutch Biomedical and Information Engineering School, Northeastern University, Shenyang City, Liaoning, Province, 110819 China; 2 Department of Cardiology, the First Hospital of China Medical University, Shenyang City, Liaoning Province, 110001 China; 3 Department of Radiology, General Hospital of Shenyang Military, Shenyang City, Liaoning Province, 110001 China; 4 Department of Biomedical Engineering, Eindhoven University of Technology, Eindhoven, 5600MB The Netherlands; 5 Department of Electrical and Computer Engineering, Stevens Institute of Technology, Hoboken, NJ07030 USA

## Abstract

Arterial stiffness is an important risk factor for cardiovascular events. Radial augmentation index (*AI*_***r***_) can be more conveniently measured compared with carotid-femoral pulse wave velocity (*cfPWV*). However, the performance of *AI*_***r***_ in assessing arterial stiffness is limited. This study proposes a novel index *AI*_***rd***_, a combination of *AI*_*r*_ and diastolic augmentation index (*AI*_*d*_) with a weight *α*, to achieve better performance over *AI*_*r*_ in assessing arterial stiffness. 120 subjects (43 ± 21 years old) were enrolled. The best-fit *α* is determined by the best correlation coefficient between *AI*_*rd*_ and *cfPWV*. The performance of the method was tested using the 12-fold cross validation method. *AI*_*rd*_ (*r* = 0.68, *P* < 0.001) shows a stronger correlation with *cfPWV* and a narrower prediction interval than *AI*_*r*_ (*r* = 0.61, *P* < 0.001), *AI*_*d*_ (*r* = −0.17, *P* = 0.06), the central augmentation index (*AI*_*c*_) (*r* = 0.61, *P* < 0.001) or *AI*_*c*_ normalized for heart rate of 75 bpm (*r* = 0.65, *P* < 0.001). Compared with *AI*_*r*_ (age, *P* < 0.001; gender, *P* < 0.001; heart rate, *P* < 0.001; diastolic blood pressure, *P* < 0.001; weight, *P* = 0.001), *AI*_***rd***_ has fewer confounding factors (age, *P* < 0.001; gender, *P* < 0.001). In conclusion, *AI*_*rd*_ derives performance improvement in assessing arterial stiffness, with a stronger correlation with *cfPWV* and fewer confounding factors.

## Introduction

Arterial stiffness is an important risk factor for cardiovascular events^1–4^ and other complications^5–7^. Many indicators have been proposed to assess arterial stiffness. Carotid-femoral pulse wave velocity (*cfPWV*) is considered the ‘gold standard’ in determining arterial stiffness^1, 8^. However, several limitations still exist. First, it is not convenient to record the carotid and femoral pulse waves simultaneously. Patients should keep in supine position. Second, the distance from the carotid to the femoral artery is difficult to measure accurately especially in patients with abdominal obesity^9^. Moreover, femoral pulse wave can not be readily and accurately measured in patients with obesity, diabetes, metabolic syndrome, or peripheral artery disease^8^.

Wave reflection, which is convenient to measure, is of great interest in the estimation of arterial stiffness, and is generally quantified by augmentation index, which is calculated from the pulse wave at a specific artery site^10–13^. Central aortic augmentation index (*AI*_*c*_) has been shown to be an independent predictor of all-cause and cardiovascular mortality in end-stage renal failure patients^10^. *AI*_*c*_ normalized for heart rate of 75 bpm (*AI*@75) has been proven to be independently associated with severe short- and long-term cardiovascular events in patients undergoing percutaneous coronary interventions^11^. However, *AI*_*c*_ can not be readily obtained non-invasively. Recent studies^12–18^ on the estimation of aortic pulse wave using transfer functions provide an alternative method to predict *AI*_*c*_ based on peripheral pulse waves. Yet, Millasseau^19^ concluded that radial augmentation index (*AI*_*r*_) provides similar information on central arterial stiffness as *AI*_*c*_ obtained by a transfer function method. *AI*_*r*_ can be directly calculated from a radial pulse wave. It is used to assess arterial stiffness in a widely used device, HEM9000AI (Omron Healthcare, Japan). Kohara^20^ showed the feasibility of *AI*_*r*_ in assessing vascular aging. *AI*_*r*_ is also reported to be a predictor of premature coronary artery disease in younger males^21^. However, the performance of *AI*_*r*_ in assessing arterial stiffness is limited, as *AI*_*r*_ is influenced by several factors other than *cfPWV*, like heart rate (HR) and the reflect distance of the pulse wave^22^. In addition, it has been shown that *AI*_*r*_ does not correlate closely with vascular stiffness in those over the age of 55^23^. Due to the limitations of *AI*_*r*_ and the fact that diastolic augmentation index (*AI*_*d*_) also reflects wave reflection^24–26^, we propose a novel index *AI*_*rd*_ in the form of a linear combination of *AI*_*r*_ and *AI*_*d*_ to derive potentially better performance over *AI*_*r*_ in assessing arterial stiffness. Our contribution include the proposed index *AI*_*rd*_ and the validation of the linear combination of *AI*_*r*_ and *AI*_*d*_, instead of *AI*_*r*_, in assessing arterial stiffness.

The subsequent contents of this paper are organized as follows. The second section describes the methodologies used in this study. The third section presents the results. The discussion and conclusion of our study are presented in the fourth and fifth sections.

## Methods

### Subjects and study protocol

128 subjects participated in the study. 8 of them were excluded for lack of accuracy in the measurement of *cfPWV*, resulting in a sample of 120 subjects (54 females, 66 males) aged 18 to 92 years old (mean ± SD, 43 ± 21 years old). 4 subjects had arrhythmias, 2 had premature ventricular contractions, and 5 had hypertension and arrhythmia, hypertension, hypothyroidism, arteriosclerosis, and mitral regurgitation, respectively. Information on the subjects is shown in Table 1 and is also detailed in Supplementary Table S1. All subjects gave informed consents before the study. The datasets generated during the current study are available from the corresponding author on reasonable request. This study was approved by School of Sino-Dutch Biomedical and Information Engineering, Northeastern University, China. The experiment was carried out in accordance with the Interim Measures for Guidelines on Ethical Review of Biomedical Research Involving Human Subjects.

**Table 1:** Information of the subjects(*n* = 120). Cf-distance: distance from the carotid to the femoral artery.

Physiological parameters	Mean ± SD	Range
Age (year)	43 ± 21	[18, 92]
Height (cm)	168 ± 8	[150, 189]
Weight (kg)	65 ± 11	[44, 95]
BMI (kg/m^2^)	23 ± 3	[17, 33]
HR (bpm)	68 ± 10	[45, 97]
SBP (mmHg)	119 ± 15	[90, 156]
DBP (mmHg)	74 ± 10	[52, 110]
Cf-distance (cm)	61.1 ± 4.5	[51, 71]

Measurements were performed in a quiet room at a constant temperature of 22 to 23 °C. Subjects stayed in supine position throughout the experiment and were advised to keep still without talking, laughing or sleeping. Subjects had a 15 min rest before the test. Measurements of augmentation indexes and *cfPWV* were performed sequentially. There was no significant difference (paired t-test: mean ± SD, −0.6 ± 3.6 bpm; *P* = 0.07) in pulse rate between the two measurements.

### Measurement of cfPWV

*cfPWV* is defined as pulse traveled distance divided by pulse transit time (PTT) from carotid to femoral artery. The pulse travelled distance was calculated as 0.8 times the direct distance from the right common carotid artery to the right common femoral artery^22^. The distance was measured using a non-elastic tape. PTT was calculated as time difference between the feet of pulse waves at two different artery sites. In each trial, right carotid and right femoral pulse waves were measured using two pressure pulse sensors (MP100, Xinhangxingye Co. Ltd., Beijing, China). The signals were recorded simultaneously for 30 seconds in each trial and were sampled at a rate of 1000 Hz.

The pulse wave signals were then pre-processed to eliminate baseline drift and noise, which influence the accuracy of subsequent calculations. Baseline drift is mainly due to body motion artifact and respiration. The baseline drift was removed by applying ‘sym7’ wavelet decomposition^27, 28^ at level 10 to the data and eliminating the approximation coefficients in the wavelet decomposition. Similarly, the noise was removed by applying ‘db7’ wavelet decomposition^27, 28^ at level 4 to the data and eliminating the detail coefficients in the wavelet decomposition.

The foot of a pulse wave was extracted using an intersecting tangents technique^29–31^, which determines the foot by the intersection of the horizontal line through the minimum and the tangent line through the maximum first derivative with respect to time.

PTT was obtained from every cardiac cycle in a series of data, and those exceeding 90% of the SD distribution curve of the PTTs were discarded. The remaining PTTs were averaged. Two measurements of *cfPWV* were applied in each subject. If the difference between two successive measurements in one subject was less than 0.5 m/s^22^, the mean of the two measurements was taken. Otherwise, the data of this subject was discarded. According to this criterion, 8 subjects were excluded as mentioned earlier.

### Pulse wave analysis

The radial pulse wave was recorded using a SphygmoCor device (AtCor, Australia) with a sampling rate of 128 Hz. The quality of the measurement was controlled by an operator index assessed by the device. A measurement that yields an operator index of lower than 85% was discarded and another measurement was performed. Two trials with an operator index higher than 85% were required on each subject, and two to five measurements were applied to achieve this goal. Augmentation indexes were calculated as the mean of the two measurements. For each measurement, an average radial pulse wave was derived using an ensemble average method. *AI*_*r*_ and *AI*_*d*_ were both calculated from the average pulse wave.

**Figure 1:**
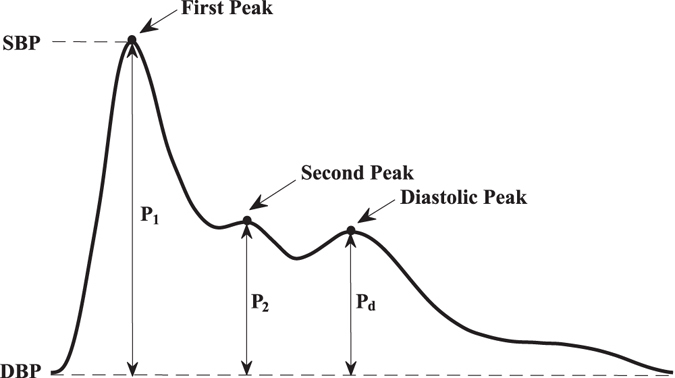
Features of the radial pulse wave. Amplitude of the peak and foot are the systolic (SBP) and diastolic (DBP) blood pressures, respectively. *P*_1_ indicates the difference between the first peak and the foot in amplitude; *P*_2_ is the amplitude of the second peak minus DBP; *P*_*d*_ is the amplitude of the diastolic peak minus DBP.

As shown in Fig. 1, *AI*_*r*_ is defined as the amplitude difference (*P*_2_) between the second peak and the foot divided by the amplitude difference (*P*_1_) between the first peak and the foot. *AI*_*d*_ is the amplitude difference (*P*_*d*_) between the diastolic peak and the foot divided by *P*_1_. The locations of the second peak and diastolic peak of all subjects were determined through a second-derivative method.

In this paper, a linear combination of *AI*_*r*_ and *AI*_*d*_ is defined as:

1 $$A{I}_{rd}=\alpha \times A{I}_{r}-(1-\alpha )\times A{I}_{d}$$

where *α* determines the weights of *AI*_*r*_ and *AI*_*d*_ in the combination. *AI*_*rd*_ equals −*AI*_*d*_ and *AI*_*r*_ when *α* is 0 and 1, respectively. *AI*_*c*_ and *AI*@75 were also included in the study for comparison with *AI*_*rd*_ in assessing arterial stiffness. *AI*_*c*_ is defined as the ratio of the late systolic boost in the aortic pressure wave and pulse pressure^32^. Both *AI*_*c*_ and *AI*@75 were calculated using the SphygmoCor device based on the central aortic pulse wave, which was estimated by applying a transfer function to the radial pulse wave.

### Statistical analysis

The reliability of all measurements were evaluated by two-way random average-measure intra-class correlation coefficients (ICC). An ICC higher than 0.9 was deemed appropriate^33^.

A 12-fold cross validation was used in the determination of *α*. The raw data was randomly grouped into 12 subsets (with 10 subjects in each). The 12 subsets were divided in all possible ways (12 in total) into a training group with 11 subsets and a test group with 1 subset. In each trial, the best-fit *α* was calculated based on the training data, and was then used to calculate *AI*_*rd*_ of the test group. The best-fit *α* was determined by finding the strongest correlation between *AI*_*rd*_ and *cfPWV*. The stability of the best-fit *α* was assessed by analysis of variance in 12 trials.

The correlation of *cfPWV* with each augmentation index (*AI*_*r*_, *AI*_*d*_, *AI*_*rd*_, *AI*_*c*_ or *AI*@75) was calculated. Prediction interval^34, 35^ was calculated to evaluate the estimate of *cfPWV* by each augmentation index. The dependence of *AI*_*r*_ and *AI*_*rd*_ were studied by performing stepwise multi-regression analysis (enter if *P* < 0.01, remove if *P* > 0.01) with the following parameters: gender, age, height, weight, HR, brachial systolic (SBP) and diastolic (DBP) blood pressure. In this study, all statistical significance tests are two-tailed. A probability value of *P* < 0.01 is considered statistically significant.

## Results

### Reliability test

The two-way random average-measure ICC of *cfPWV* (*n* = 120) is 0.99 (*P* < 0.001). The ICCs of *AI*_*r*_, *AI*_*d*_, *AI*_*c*_ and *AI*@75 (*n* = 120) are 0.99 (*P* < 0.001), 0.95 (*P* < 0.001), 0.98 (*P* < 0.001) and 0.98 (*P* < 0.001), respectively. All the measurements in this study derive an ICC higher than 0.9.

### Regression analysis

**Figure 2:**
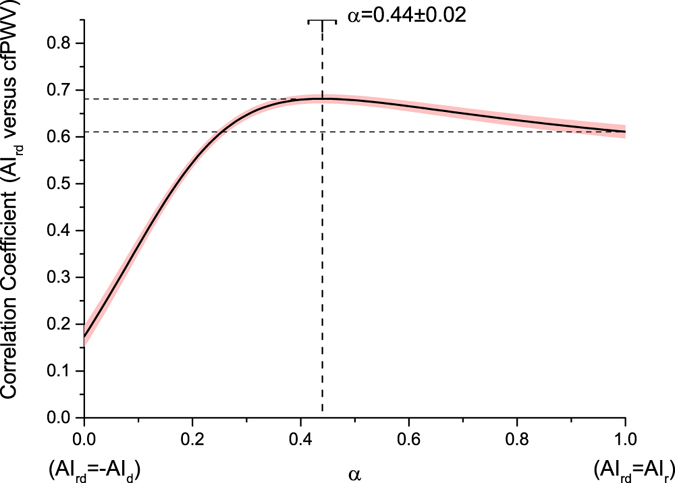
Determination of *α*. The solid line and the dashed area indicate the correlation coefficients between *AI*_*rd*_ and *cfPWV* with the change of *α*. The solid line is the mean in all 12 trials and the dashed area the confidence band. The best-fit *α* was determined by the peak of the correlation coefficient curve in each trial. The vertical dash line indicates the mean of the best-fit *α* in 12 trials, and the bar indicates the standard deviation.

Figure 2 shows the determination and stability analysis of *α* in 12 trials. The correlation coefficient between *AI*_*rd*_ and *cfPWV* is stable and so is the best-fit *α*, which is determined with respect to the peak of each correlation coefficient curve. The mean ± SD of all best-fit *α* in the 12 trials is 0.44 ± 0.02. Thus, *α* was determined as 0.44. When *α* equals 0.44, the correlation coefficient of *AI*_*rd*_ with *cfPWV* improves by 0.07 ± 0.01, compared with that of *AI*_*r*_ with *cfPWV*.

**Figure 3:**
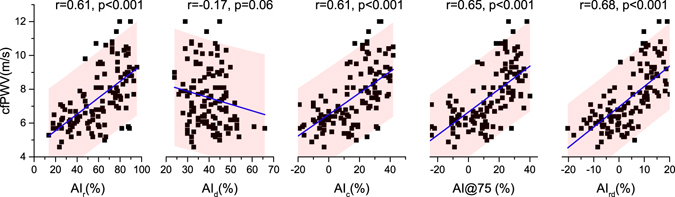
Regression analysis (*n* = 120): linearity of *cfPWV* with *AI*_*c*_, *AI*@75, *AI*_*r*_, *AI*_*d*_ and *AI*_*rd*_. Solid lines are the regression lines. The shaded areas indicate the 95% prediction interval.

Regression analysis (*n* = 120) between *cfPWV* and each augmentation index is shown in Fig. 3. *cfPWV* shows a stronger correlation with *AI*_*rd*_ (*r* = 0.68; *P* < 0.001) than with *AI*_*r*_ (*r* = 0.61; *P* < 0.001), *AI*_*c*_ (*r* = 0.61; *P* < 0.001), or *AI*@75 (*r* = 0.65; *P* < 0.001). No significant correlation between *cfPWV* and *AI*_*d*_ (*r* = −0.17; *P* = 0.06) was found. In addition, compared with other augmentation indexes, *AI*_*rd*_ derives a narrower prediction interval in the estimation of *cfPWV*.

Multi-regression analysis (*n* = 120) shown in Table 2 reveals that *AI*_*r*_ is significantly associated with age (*P* < 0.001), gender (*P* < 0.001), HR (*P* < 0.001), DBP (*P* < 0.001), and weight (*P* = 0.001). *AI*_*d*_ is significantly dependent on HR (*P* < 0.001), DBP (*P* < 0.001), and age (*P* = 0.001). *AI*_*rd*_ is only associated with age (*P* < 0.001) and gender (*P* < 0.001).

**Table 2:** Multi-regression analysis (stepwise, enter if *P* < 0.01, remove if *P* > 0.1) for *AI*_*r*_ and *AI*_*rd*_ (*n* = 120).

Dependants	Variables	*β*	*t*	*P*
*AI*_*r*_ (%)	Age	0.657	12.039	<0.001
	Gender	0.254	4.018	<0.001
	HR	−0.312	−5.443	<0.001
	DBP	0.307	4.971	<0.001
	Weight	−0.219	−3.353	0.001
*AI*_*d*_ (%)	HR	−0.742	−10.979	<0.001
	DBP	0.320	4.696	<0.001
	Age	−0.318	−4.692	<0.001
	Height	−0.237	−3.454	0.001
*AI*_*rd*_ (%)	Age	0.789	16.305	<0.001
	Gender	0.298	6.167	<0.001

*β* is the regression coefficient. *t* is the t-value for each individual *β*.

## Discussion

The significance of *AI*_*r*_ has been presented in multiple studies^20, 21^. However, the performance of *AI*_*r*_ in assessing arterial stiffness is unsatisfactory^22, 23^. The present study proposed a novel index, *AI*_*rd*_, by combining *AI*_*r*_ and *AI*_*d*_ with a weight coefficient *α*. The weight *α* is stable in 12 trials. *AI*_*rd*_ correlates better with *cfPWV* compared with *AI*_*r*_, *AI*_*d*_, *AI*_*c*_ and *AI*@75, and is dependent on fewer confounding factors than *AI*_*r*_.

The best-fit *α* is stable in 12 trials (mean ± SD, 0.44 ± 0.02). The mean best-fit *α* derives stable improvement of *AI*_*rd*_ over *AI*_*r*_ in assessing arterial stiffness (with the improvement in correlation coefficient of *AI*_*rd*_ over *AI*_*r*_ with *cfPWV* being 0.07 ± 0.01 when *α* = 0.44 in the training data of 12 trials). In addition, in Fig. 2, a wide range of *α* (from 0.25 to 1.0) allows *AI*_*rd*_ better performance over *AI*_*r*_. The stability and this wide range of *α* demonstrates the reliability and feasibility of the proposed method.

As central arteries become stiffer, *cfPWV* increases and the reflected wave from lower body returns to the ascending aorta earlier and also arrives at the radial artery earlier, which causes increases in both *AI*_*c*_ and *AI*_*r*_^36, 37^. Thus, both *AI*_*c*_ and *AI*_*r*_ reflect central arterial stiffness, which is demonstrated in the present study (with the correlation coefficient between *AI*_*r*_ and *cfPWV*, *r* = 0.61; *P* < 0.001; and the correlation coefficient between predicted *AI*_*c*_ and *cfPWV*, *r* = 0.61; *P* < 0.001), and also in multiple previous studies^20, 37, 38^. Millasseau *et al*.^19^ further concluded that *AI*_*r*_ provides similar information on central arterial stiffness as *AI*_*c*_ obtained by applying a transfer function to the radial pulse wave (*AI*_*r*_ versus *AI*_*c*_, *r* = 0.94, *P* < 0.001). Similar results were derived in Kohara’s study^20^, and also in the present study with a significant correlation between *AI*_*r*_ and *AI*_*c*_ (*r* = 0.95, *P* < 0.001). *AI*_*c*_ directly measured in the aorta might derive a stronger correlation with *cfPWV*. However, the aortic pulse wave cannot be readily acquired directly using noninvasive techniques. The most commonly used noninvasive technique is to apply a generalized transfer function^12, 13^ to the radial pulse wave, which derives satisfactory performance in the estimation of central aortic blood pressures. Specialized transfer function techniques^14–18^ proposed in recent years further improve the accuracy. However, these techniques are unable to derive satisfactory performance in predicting *AI*_*c*_. The reason is that the accuracy of the inflection point, based on which *AI*_*c*_ is calculated, depends on higher frequency components of the aortic pulse wave, which are difficult to obtain accurately from the transfer function, either generalized or specialized. *AI*_*c*_ predicted by individualized transfer functions is a promising approach to assess arterial stiffness, however, its accuracy requires further improvements.

*AI*_*rd*_ (*r* = 0.68; *P* < 0.001) correlates better with *cfPWV* than *AI*_*r*_ (*r* = 0.61; *P* < 0.001) does, with a narrower prediction interval. *AI*_*r*_ is not only determined by *cfPWV*, but is also influenced by HR^39, 40^ (inversely) and the changes in reflection sites at the lower body^8^. The reflecting site distance from the aorta is related to reflected wave amplitude^41^, which is equal to or largely contributes to the amplitude of diastolic peak. HR inversely influences DBP^42^. DBP affects reflecting site distance^8^ and peripheral resistance^41^, both of which are determinants of reflected wave amplitude and also the amplitude of diastolic peak. The weighted subtraction of *AI*_*d*_ from *AI*_*r*_ could reduce the influence of changes in reflection sites on *AI*_*r*_. This can be demonstrated through our result that *AI*_*r*_ and *AI*_*d*_ both significantly correlate with DBP(*P* < 0.001 for both) and HR (*P* < 0.001 for both), while *AI*_*rd*_ shows no significant correlation with DBP or HR.

The multi-regression analysis (Table 2) demonstrated that *AI*_*r*_ is dependent on factors including age (*P* < 0.001), gender (*P* < 0.001), HR (*P* < 0.001), DBP (*P* < 0.001), and weight (*P* = 0.001). This is consistent with previous studies by Sugawara *et al*.^43^ and Kohara *et al*.^20^. *AI*_*d*_ is significantly correlated with HR (*P* < 0.001), DBP (*P* < 0.001), and age (*P* = 0.001). *AI*_*rd*_ is only associated with age (*P* < 0.001) and gender (*P* < 0.001). This means that by linearly combining *AI*_*r*_ with *AI*_*d*_, the influence of DBP and HR is reduced, which allows *AI*_*rd*_ a higher reliability and better applicability than *AI*_*r*_ in assessing arterial stiffness.

Our study has a few limitations. During the experiment, all subjects were required to be in supine position. The stability of *α* and the performance of *AI*_*rd*_ in assessing arterial stiffness in other postures (for instance, sitting) is not evaluated. Besides, differences in *AI*_*r*_ could exist when measuring radial pulse wave using different devices^44^. The best-fit *α* might also be different when *AI*_*r*_ and *AI*_*d*_ were measured using different devices.

## Conclusion

In conclusion, *AI*_*rd*_ derives performance improvement over *AI*_*r*_ in assessing arterial stiffness, with stronger correlation with *cfPWV* and fewer confounding factors. *AI*_*rd*_ is a potential surrogate for both central and radial augmentation indexes in assessing arterial stiffness, with the same measurement procedure but achieving improved performance. Comparing to the ‘gold standard’, *cfPWV*, methods based on pulse wave analysis (*AI*_*r*_ and *AI*_*rd*_) are much more convenient in the assessment of central arterial stiffness. However, in order to evaluate the physiological and pathological significance of *AI*_*rd*_, longitudinal studies are needed on the relationship between *AI*_*rd*_ and cardiovascular events.

## Electronic supplementary material

Supplementary Dataset
